# mSphere of Influence: Coordinating effective clearance of bacterial bloodstream infections

**DOI:** 10.1128/msphere.00521-23

**Published:** 2023-10-24

**Authors:** Caitlyn L. Holmes

**Affiliations:** 1 Department of Pathology, University of Michigan Medical School, Ann Arbor, Michigan, USA; 2 Department of Microbiology and Immunology, University of Michigan Medical School, Ann Arbor, Michigan, USA; University of Michigan, Ann Arbor, Michigan, USA

**Keywords:** bloodstream infections, bacteremia, *Klebsiella*, Gram-negative bacteria

## Abstract

Caitlyn Holmes works in the field of bacterial pathogenesis and host-pathogen interactions. In this mSphere of Influence article, she reflects on how the papers “Innate Lymphocyte/Ly6C^hi^ Monocyte Crosstalk Promotes *Klebsiella pneumoniae* Clearance” by Xiong et al. (H. Xiong, J. W. Keith, D. W. Samilo, R. A. Carter, et al., Cell 165:679–89, 2016, https://doi.org/10.1016/j.cell.2016.03.017) and “Dual-Track Clearance of Circulating Bacteria Balances Rapid Restoration of Blood Sterility with Induction of Adaptive Immunity” by Broadley et al. (S. P. Broadley, A. Plaumann, R. Coletti, C. Lehmann, et al., Cell Host Microbe 20:36–48, 2016, https://doi.org/10.1016/j.chom.2016.05.023) impacted her research by highlighting the tangled web of immune responses that influence bacterial bloodstream infections.

## COMMENTARY

Sepsis is a leading cause of global morbidity and mortality, defined by organ dysfunction from a dysregulated immune response to infection (1). Bloodstream infections can initiate sepsis; thus, the immune system must clear circulating pathogens without harming the host. The host immune response can be compared to a basketball team. A coordinated group of players with unique talents is required to contain an opposing force, and a dysregulated response can send the system into chaos. Over 90% of bloodstream infections are initiated by bacteria (2, 3). Bacteremia, the presence of bacteria in the bloodstream, is usually transient, and invading species are effectively cleared by circulating immune cells and blood filtering organs (4). However, bacteria can establish systemic infection by disseminating from primary sites and colonizing tissues. Gram-negative species cause about half of bacteremia. This number is expected to rise due to the antimicrobial resistance crisis and is concerning due to the potential to initiate sepsis (5). *Klebsiella pneumoniae* is a leading cause of Gram-negative bacteremia and the focus of my research. I am fascinated by how *K. pneumoniae* survives in blood filtering organs and how the host clears this pathogen. When considering the host-pathogen intersection during *K. pneumoniae* infection, I am inspired by the work of Xiong and colleagues in the study “Innate Lymphocyte/Ly6C(hi) Monocyte Crosstalk Promotes *Klebsiella pneumoniae* Clearance” (6), where the authors defined a surprising role for innate lymphocytes in pneumonia. Similarly, the work of Broadley and colleagues in “Dual-Track Clearance of Circulating Bacteria Balances Rapid Restoration of Blood Sterility with Induction of Adaptive Immunity” (7) reminds me that immune responses in bacteremia are multi-faceted.

Monocytes are circulating innate immune cells recruited to infected tissues and can differentiate to aid in pathogen clearance or inflammation resolution. For *K. pneumoniae*, monocytes have a complex role in the clearance of pneumonia. Monocytes are critical to resolve *K. pneumoniae* lung infection, and Xiong et al. defined the downstream immune orchestration coordinated by these cells (6). Through elegant approaches including bone marrow depletion, adoptive transfers, and cytokine profiling, monocytes were defined as the predominant tumor necrosis factor alpha (TNF-α) producers during *K. pneumoniae* pneumonia. Monocyte TNF-α secretion recruited type 3 innate lymphoid cells (ILC3s) to the lung, which in turn produced IL-17A, a cytokine that protects the lung against *K. pneumoniae*. Thus, the authors defined that ILC3s work in concert with monocytes to produce a positive feedback loop that aids in *K. pneumoniae* clearance. This study is fascinating because host-pathogen interactions are often boiled down to interactions of bacteria with a single immune subset, but the authors demonstrate a more complex reality *in vivo*. While inflammatory monocytes compose a substantial portion of immune cells recruited to the lung during *K. pneumoniae* infection, their role extends beyond directly killing bacteria. As with a basketball team, there are “superstar” players of the immune system, ones whose talents are well known and appreciated (monocytes). A team cannot be composed of a singular talent as the functions of other players (innate lymphoid cells [ILCs]) are required for an effective response. All players work together to keep the system balanced. A separate study found that myeloid-derived suppressor cells, another monocyte-derived subset, secreted a soluble factor that influenced the ability of neutrophils to phagocytize *K. pneumoniae* (8). Monocytes have multiple roles during infection and work in conjunction with many cell types to elicit a tangled web of host responses that are not yet fully characterized.

After disseminating from a primary site, bacteria encounter filtering organs in the bloodstream which are equipped to clear circulating pathogens. The findings of Broadley and colleagues underscore the complexities of host-pathogen interactions during bacteremia and demonstrate that immune responses are not uniform in systemic infection (7). Using intravital microscopy, which allowed for visualization of circulating bacteria, the authors found two types of responses that occurred simultaneously. In a “fast track” response, immune cells in the liver aided in the quick destruction of circulating pathogens. In a “slow track” response, phagocytes in the spleen assisted with an adaptive response to pathogens prior to their clearance. Thus, multiple immune reactions can occur with different purposes across a system. In a truly compelling experiment, the authors extended these findings across species. Multiple bacteria, including *K. pneumoniae*, exhibited patterns of fast and slow clearance in bloodstream infection. This study is another example of how host-pathogen interactions extend beyond a direct killing mechanism and underscore the multi-faceted nature of immune responses. In basketball, there are two methods for building a team. The first is to recruit many superstars at once, like “fast” track bacteremia clearance. While this is an effective short-term strategy, it does not protect from long-term damage. Alternatively, “slow” track team building shapes a successful program over time, like adaptive immunity, which supports protection against repeated pathogen exposure. The best teams blend support of their superstars with an investment in team cohesiveness using fast and slow tracks. In bacteremia, it is critical to understand both pathways as they may present opportunities for novel immunotherapies. For example, targeting fast clearance pathways may enhance pathogen destruction, while supporting slow clearance pathways may prevent recurrent infection.

Reflecting on these studies, I am inspired by the consideration that pathogen clearance is multi-faceted and the result of many cell types. These articles have challenged me to consider the role of multiple immune subsets during Gram-negative bacteremia. When investigating *K. pneumoniae* bacteremia, I consider how immune subsets work together and how populations with relatively small abundance can have a high impact on immunity. Since bacteria encounter multiple tissues during systemic infection, there are also many host-pathogen interfaces. At each one, host cells work in concert to clear infection and resolve inflammation. Bacteria like *K. pneumoniae* must employ distinct strategies to resist immune responses in different tissues. Given the number of immune subsets, infected tissues, and bacterial species, there is much left to learn! Xiong et al. and Broadley et al. have highlighted the web of immunity in bacteremia, and their work has inspired my goal to understand interactions between immune cells and bacteria during systemic infection.
